# Targeted inhibition of MEK1 by cobimetinib leads to differentiation and apoptosis in neuroblastoma cells

**DOI:** 10.1186/s13046-015-0222-x

**Published:** 2015-09-18

**Authors:** Anjali Singh, Yibing Ruan, Tanya Tippett, Aru Narendran

**Affiliations:** POETIC Laboratory for Preclinical and Drug Discovery Studies, University of Calgary, Calgary, Alberta Canada; Division of Pediatric Oncology, Alberta Children’s Hospital, 2888 Shaganappi Trail NW, Calgary, Alberta T3B 6A8 Canada; Memorial Sloan-Kettering Cancer Center, 1275 York Avenue, New York, NY 10065 USA

**Keywords:** Neuroblastoma, Cobimetinib, Apoptosis, Differentiation, Targeted therapy

## Abstract

**Background:**

Neuroblastoma (NB) is one of the most common childhood malignancies. Currently, high risk NB carries a poor outcome and significant treatment related toxicities and, thus has been a focus for new therapeutics research in pediatric oncology. In this study, we evaluated the effects of the MEK inhibitor cobimetinib, as a single agent and in combinations, on the growth, survival and differentiation properties against a molecularly representative panel of NB cell lines.

**Methods:**

*In vitro* anti-proliferative activity of cobimetinib alone or in combination was investigated by cell viability assays and its target modulatory activity was evaluated using phospho-kinases antibody arrays and western blot analysis. To determine the effect of combination with cis-RA on differentiation and resulting enhanced cellular cytotoxicity, the expression of glial fibrillary acidic protein (GFAP) and microtubule-associated protein 2 (MAP2) expression levels were examined by immuno-fluorescence.

**Results:**

Our findings show that cobimetinib alone induced a concentration-dependent loss of cell viability in all NB cell lines. In addition, cobimetinib showed feedback activation of MEK1/2, and the dephosphorylation of extracellular signal-regulated kinases (ERK1/2) and c-RAF, providing information on the biological correlates of MEK inhibition in NB. Combined treatment with cis-RA, led to differentiation and enhanced sensitization of NB cells lines to cobimetinib.

**Conclusion:**

Collectively, our results provide evidence that cobimetinib, in combination with cis-RA, represents a feasible option to develop novel treatment strategies for refractory NB.

## Background

Neuroblastoma (NB) is a malignancy of the embryonal sympathetic nervous system arising from neuroblasts and is the most common type of solid tumors in children [1]. Although cure rates have improved over the past 20 years, event-free survival is still only about 45 % for patients with high-risk metastatic disease [2]. Heterogeneity is the hallmark of NB and its clinical behavior ranges from spontaneous regression to metastatic disease that is refractory to common therapies. Advanced NB typically metastasizes to regional lymph nodes, bone marrow, skin and liver [3]. Distinct prognostic stages (1, 2A, 2B, 3, 4 and 4S) have been identified for the classification of NB [4]. The best-characterized genetic alterations of NB include N-Myc oncogene- amplification or allelic loss, near triploid karyotype, deletion of short arm of chromosome 1, chromosomal rearrangements involving chromosome 11q and high expression of tropomyosin receptor kinase A (TrkA) and B (TrkB). To improve the clinical outcome of advanced NB, it is important to identify the key molecularly defined actionable pathways and targets for novel therapeutics in these patients.

The Mitogen-activated protein kinases (MAPKs) cascade (Ras/Raf/MEK/ERK) is an important signal transduction system involved in the control of cell proliferation, survival and differentiation [5]. A wide range of cell-surface molecules activate RAS (KRAS, NRAS, and HRAS), a family of GTPases, that act as a molecular switch in the activation of MAPKs cascade (Raf/MEK/ERK) [6]. There are multiple molecular mechanisms of interaction and activation between the upstream nodes of the Ras/Raf/MEK/ERK cascade and other cell signaling pathways, ultimately resulting in ERK transcription factor activation [7]. The activation of ERK leads to cells acquiring many of the hallmarks of cancer such as cell survival, cell migration, and invasion and inhibitors targeting this pathway have been vigorously developed [8, 9]. The MEK inhibitors act on MEK phosphorylation by binding to a pocket adjacent to the ATP binding site, decreasing both the amount of MEK activity, and the quantity of activated ERK in the cell. Cobimetinib is a potent and highly selective inhibitor of MEK1 [10].

In this study, we evaluated the expression and activity of MEK and ERK in a panel of NB tumor cell lines and their sensitivity to cobimetinib *in vitro*. The panel of cell lines used included the neuroblastic (N), substrate-adherent (S), and intermediate (I) subtypes, classified based on their morphology, growth patterns, and malignant potential [11]. In addition, in drug combination studies we have also tested the ability of cis-retinoic acid (cis-RA) that has been known to cause cell growth inhibition and differentiation [12], to enhance the activity of cobimetinib against NB cells. Our data provide initial proof-of-concept information on the potential utility of cobimetinib as an effective targeted therapeutic agent against refractory NB.

## Materials and methods

### Cell lines and cell culture

The following NB cell lines were used: SK-N-AS (ATCC-CRL-2137), SK-N-SH (ATCC HTB-11), SK-N-BE(2) (ATCC CRL- 2271), IMR-32 (ATCC CCL-127), SHEP, IMR-5. SHEP and IMR-5 cell lines were a gift from Dr. Herman Yeger (The hospital for Sick Children, Toronto, ON). These cells were maintained in Opti-MEM media (Gibco, Invitrogen Corporation, Burlington, ON) supplemented with 5 % fetal bovine serum and 100 units/ml penicillin and 100 units/ml streptomycin (Gibco). Confluent cells were trypsinized with 0.25 % Trypsin-EDTA in Ca^2+^ and Mg^2+^ free balanced salt solution (Gibco) every three to five days. All cell cultures were maintained in incubators at 37 °C in a humidified atmosphere with 5 % CO_2._

The MEK inhibitor cobimetinib (GDC-0973) was kindly provided by Roche (Basel, Switzerland).

Stock solutions of cobimetinib were prepared as 10 mM in DMSO and stored in aliquots at −20 °C. Cis-RA was obtained from Sigma (Oakville, ON).

### Drug cytotoxicity assays

Neuroblastoma cells were trypsinized and placed in 96 well plates (Grenier Bio One, Monroe, NC) at a concentration of 5 x 10^3^ cells per well. Increasing concentrations of study agents and a corresponding dilution of DMSO were added to a final volume of 200 μl per well. After four days in culture, cell survival was quantified by an inverted microscope (Cyntellect Inc, San Diego, CA; http://www.nexcelom.com/Celigo/direct-cell-counting-assays-for-immunotherapy.php#feature6). The half maximal inhibitory concentration (IC_50_) values were calculated for each agent based on individual cytotoxicity plots.

### Human phospho-kinase antibody array

Neuroblastoma cells were seeded in six well culture plates (Nunc, Waltham, MA) at 1 x 10^6^ cells/ml and incubated overnight. Fresh culture medium containing cobimetinib or vehicle control was added and after two hour incubation, cells were washed with ice cold PBS and treated with lysis buffer (50 mM Tris, 5 mM EDTA, 0.1 % SDS, 1 % Triton X-100, 0.5 % sodium deoxycholate) containing phosphatase and protease inhibitors (Sigma). Human phopsho-kinase array (R&D Systems, Inc., Minneapolis, MN) were incubated with cobimetinib-treated and control cell lysates (150 μg) over-night, washed and probed with horseradish peroxidase (HRPO) (Sigma) labeled anti-pTyr antibodies according to manufacturer’s protocol. The arrays were scanned and the spot densities were quantified with ImageJ (National Institutes of Health, http://rsb.info.nih.gov/ij/ version *1.4.3.67*).

### Western blot analysis for protein and phosphoprotein detection

Each NB cell line was grown to 70 to 80 % confluence in six well culture plates (Nunc) and incubated overnight to allow for cell adherence. The cells were then incubated with fresh culture medium containing cobimetinib or vehicle control as indicated in individual experiments. After each time period incubation, cells were washed with ice cold PBS and treated with lysis buffer containing phosphatase and protease inhibitors. Protein concentrations of the lysates were quantified by BCA Protein Assay (Pierce, Rockford, IL). Proteins were then separated on a 8 % polyacrylamide gel electrophoresis and transferred onto nitrocellulose (NC) membranes (Bio-Rad, Mississauga, ON). The membranes were blocked for one hour at room temperature with 5 % skim milk powder in PBS containing 0.1 % Tween-20 (Sigma). The blots were incubated with primary antibodies (Cell Signaling Technology, Danvers, MA) overnight at 4 °C, washed and probed with appropriate secondary antibodies conjugated to horseradish peroxidase (HRPO) (Sigma), followed by a luminal based substrate (Mandel, Guelph, ON) and developed by exposure to x-ray film (Fisher Scientific, Ottawa, ON).

### Annexin V staining for apoptosis

Neuroblastoma cell lines (IMR-32, SHEP and IMR-5) were plated at a concentration of 3 × 10^5^ cells per well. Following treatment with 1 μM cobimetinib or DMSO (vehicle control) the cells were incubated for a period of 24 hour prior to FACS analysis. Apoptosis was measured using the Annexin V-FITC Apoptosis Detection kit (Life Technologies, Carlsbad, CA) according to the manufacturer’s instructions. In this assay, the apoptotic cells were differentiated from viable or necrotic cells by the combined application of Annexin V-FITC and propidium iodide (PI). Briefly, the control and treated cells (1 × 10^6^) were re-suspended in 500 μl of binding buffer and incubated with 5 μl of Annexin V-FITC and 1 μl of PI solution for 15 min. The samples were then analyzed on a BD Facscan Instrument (BD Biosciences, Franklin Lakes, NJ), measuring the Annexin V-FITC emission at 488 nm and PI emission at 575 nm. Lower right quadrant (Q4) represent percentage of early apoptotic cells out of total cell population in the treatment group compared to control.

### Treatment with cis-RA

Cis-Retinoic acid was added to a final concentration of 10 μM, according to feasible pharmacological dosages and those used in previous *in vitro* differentiation studies [13, 14]. To see the combined effect of cis-RA and cobimetinib on cell growth inhibition, IC_25_ concentration of cobimetinib (i.e., the amount that induced 25 % cell death in single drug studies) was added to cultures containing increasing concentrations of cis-RA. The number of viable cells present after four days in culture was determined as described.

### Immunocytochemical detection of differentiation markers

Neuroblastoma cells were treated with cobimetinib (1 μM) and cis-RA (10 μM) alone or in combination for 24 hours. Briefly, the cells were fixed with 4 % paraformaldehyde (Sigma) and permeabilized with 0.05 % Triton X-100 (Sigma). The cells were incubated with antibodies to Nestin (R&D Systems, 1:1000), GFAP (Sigma, 1:1000) and MAP-2 (Sigma, 1:800) for two hours at 37 °C. The cells were then washed with PBS and incubated with fluorescence labelled secondary antibody (Invitrogen, 1:500) at room temperature for one hour. Staining of treated and untreated cells were then visualized by fluorescence microscopy for detection of differentiation markers.

### Statistical analysis

For 2-group comparisons, Student *t* tests using the GraphPad Prism software (version 4.0) were used. The results are considered statistically significant versus the untreated cells, with a probability level of *p* < 0.05 (*), or statistically highly significant with a probability level of *p* < 0.01 (**).

## Results

**Fig. 1:**
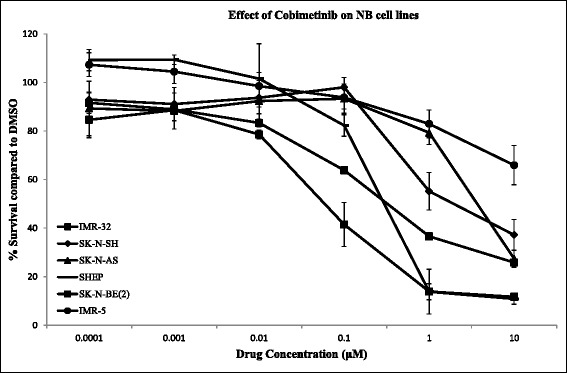
Cobimetinib mediated cytotoxicity against NB cells. Triplicate wells of cells from six NB cell lines were treated with increasing concentrations of cobimetinib or corresponding concentrations of vehicle control (DMSO) and cell viability was evaluated after four days in culture using automated microscopy. Results presented show cell growth inhibition compared to DMSO control. Data presented are representative of three separate experiments

**Fig. 2:**
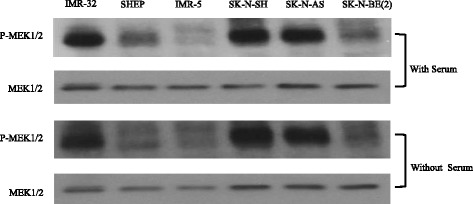
Constitutive phosphorylation status of MEK in NB cells: NB cells were grown in serum containing and serum free media and collected at their exponential growth phase (three days after sub-culture). These cells were lysed in buffer containing protease and phosphatase inhibitors and probed for total and phosphorylated MEK by western blot analysis. Data presented are representative of three separate experiments

In order to evaluate the cytotoxic effects of cobimetinib against NB, cells from a panel of cell lines were incubated with increasing concentrations of cobimetinib. After four days in culture, cell viability was evaluated. Results presented in Fig. 1 show that cell line IMR-32 is highly sensitive to cobimetinib with IC_50_ of 0.07 μM and IMR-5 was least sensitive for cobimetinib showing activity at 10 μM. Other NB cell lines fell in between this range of IC_50_ values showing an intermediate activity. In the next set of experiments, the activation status of MEK was evaluated by western blot analysis under serum containing and serum free culture conditions. Findings from these experiments show that cells with increased phosphorylated MEK were more sensitive to cobimetinib compared to those with less MEK1/2 phosphorylation (Fig. 2). There was no significant difference in MEK1/2 activity between cells grown with serum and without serum suggesting that the growth factors found in serum do not have significant influence in the base-line MEK activity found in these cells in culture.

**Fig. 3:**
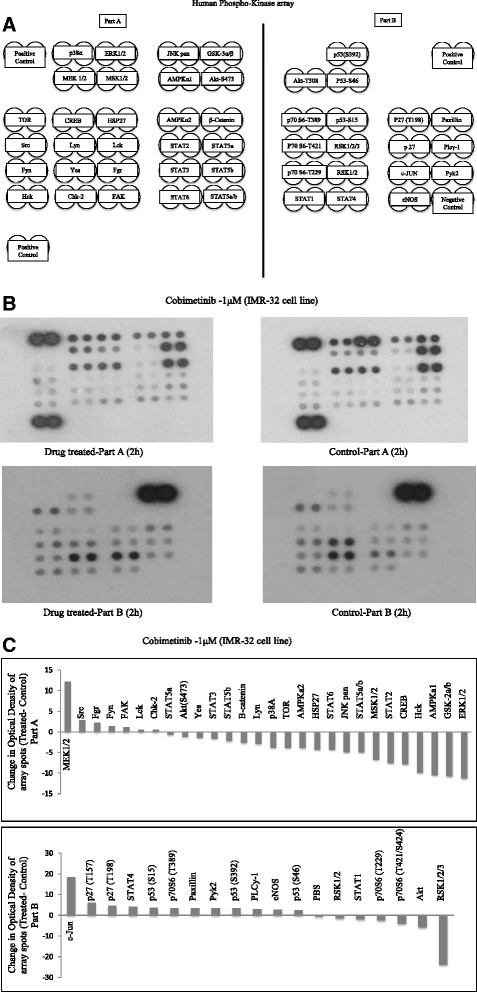
Identification of potential targets of cobimetinib activity on NB cells. Antibody arrays to a panel of signaling molecules were used to screen for dephosphorylation effects by cobimetinib. Proteins were extracted from IMR-32 cells treated with 1 μM of cobimetinib and cell lysates were made with lysis buffer containing protease and phosphatase inhibitors. DMSO treated cells were used as control. Antibody arrays were incubated with 200 μg of proteins over night at 4 °C with gentle mixing and probed with HRPO conjugated anti-phospho Tyr/Ser/Thr antibodies as per manufacturer’s protocol. Presented is the map of the orientation of the antibodies on the array (**a**), luminographically developed blots of cobimetinib treated and DMSO treated blots (**b**) and the waterfall plot showing changes in phosphorylation status of each signaling molecule on the arrays (**c**). Findings were representative of two completely separate experiments

Next, we wanted to evaluate the target modulatory activity of cobimetinib in NB cells. Antibody arrays to a panel of signaling molecules were used to screen for phosphorylation effects by cobimetinib. Proteins were extracted from cells treated with 1 μM of cobimetinib and cell lysates were made with lysis buffer containing protease and phosphatase inhibitors. DMSO treated cells were used as control. Antibody arrays were incubated with 200 μg of proteins over night at 4 °C with gentle mixing and probed with HRPO conjugated anti-phospho antibodies. Figure 3a provides a map of the positions of the various kinases on the arrays used. Figure 3b shows changes in phosphorylation as seen by gain or loss of signal on the x-ray film. A waterfall graph showing the change in optical density of array spots of cobimetinib treated cells compared to control DMSO treated cells is given in Fig. 3c. As can be seen in these figures, there is a gain in MEK phosphorylation whereas a loss in phosphorylation was noticed in ERK1/2. In addition, c-Jun also showed a gain in phosphorylation while a decrease in phosphorylation was noted with RSK1/2/3.

**Fig. 4:**
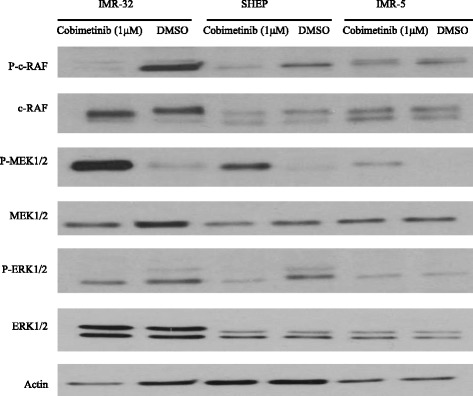
Effect of cobimetinib on MAPK cascade pathway. Changes in activation status of RAF, MEK and ERK were evaluated in three NB cell lines after treatment with cobimetinib (1 μM) or DMSO for four hours. Cell lysates were made with lysis buffer containing protease and phosphatase inhibitors and probed for total and phospho specific antibodies by western blot analysis. Data presented is representative of three separate experiments

The antibody array findings were then confirmed using western blot analysis (Fig. 4). Three NB cell lines IMR-32, SHEP and IMR-5 representing sensitive, intermediate and resistant cell toxicity against cobimetinib respectively were used for western blot analysis. The NB cell lines were incubated with cobimetinib (1 μM) or corresponding DMSO controls for four hours, harvested and subjected to western blotting. Results showed cobimetinib treatment induced de-phosphorylation of c-RAF and ERK and an increase in the phosphorylation of MEK.

**Fig. 5:**
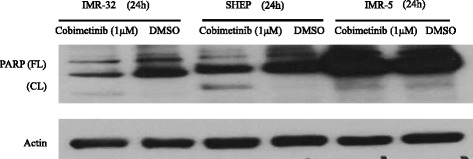
PARP cleavage induced by cobimetinib. Neuroblastoma cell lines IMR-32, SHEP and IMR-5 were incubated in the absence or presence of cobimetinib (1 μM) for 24 hour and cell lysates were analyzed by western blotting using antibodies specific for PARP and its cleaved fragment. Actin was used as loading control. Data presented is representative of two separate experiments

**Fig. 6:**
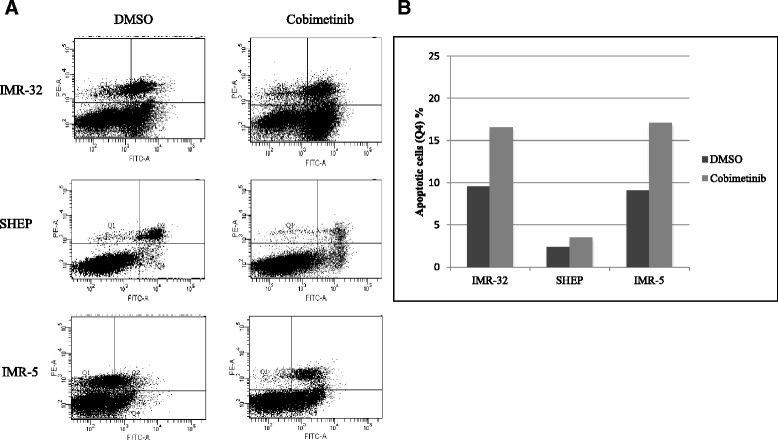
The flow cytometric analysis of apoptosis in NB cells using FITC-annexin V and PI double staining. (**a**) Apoptosis was evaluated after treating NB cells with 1 μM of cobimetinib for 24 hour, and staining with Annexin-V. Annexin V+/PI− (lower right quadrant) areas represent early apoptotic cells, and Annexin V+/PI+ (upper right quadrant) areas for late apoptotic or necrotic cells. (**b**). Quantitative analysis of the percentage of early apoptotic cells in all NB cell lines. Data presented is representative of two separate experiments

The drug sensitivity studies provided evidence for cobimetinib induced reduction in viable cell numbers after exposure. To further evaluate the potential role of apoptosis in this process, we examined PARP cleavage induced by cobimetinib (Fig. 5). Neuroblastoma cell lines IMR-32, SHEP and IMR-5 were incubated in the absence or presence of cobimetinib (1 μM) for 24 hour and cell lysates were analyzed by western blot. IMR-32 and SHEP showed PARP cleavage as indication of apoptosis induction. To further confirm this finding an Annexin V/PI binding assay was performed (Fig. 6a and b). Our data revealed an increased percentage of early apoptotic cells (Annexin V+/PI−) after treatment of IMR-32 and IMR-5 cells with cobimetinib for 24 h. There was no significant difference in the percentage of early apoptotic cells between the control group and the SHEP cell line.

**Fig. 7:**
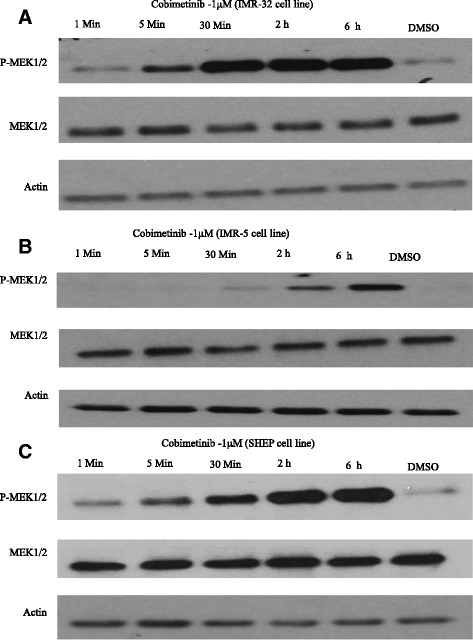
Time course analysis of cobimetinib on MAPK cascade pathway: Cells from three NB cell lines (IMR-32 (**a**), IMR-5 (**b**) and SHEP (**c**)) were harvested after treatment with 1 μM cobimetinib or DMSO control for the indicated periods. Cells were then lysed in protease and phosphatase containing buffer and probed for total and phosphorylated MEK by western blot analysis. Data presented is representative of three separate experiments

Next, studies were carried out to examine time dependent changes induced by cobimetinib with respect to some of the changes in growth regulatory pathways detected in the western blot analysis (Fig. 7a). In these experiments, IMR-32 Cells were harvested after treatment with or without 1 μM cobimetinib for the indicated periods. The activation of MEK1/2 was noticed as earlier as five minute after treatment. Data from similar analysis on IMR-5 and SHEP are presented in Fig. 7b and c respectively. Both SHEP and IMR-5 cell lines showed increase of MEK1/2 phosphorylation in the indicated time period.

**Fig. 8:**
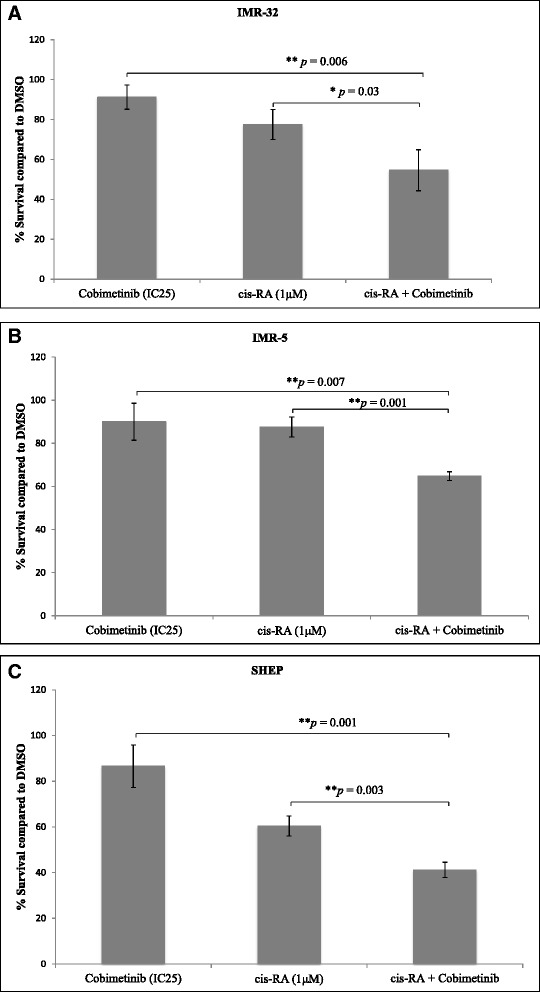
Drug combination studies of cobimetinib with cis-RA in NB cell lines: NB cells were incubated with 1 μM of cis-RA acid plus IC_25_ concentration of cobimetinib for each cell line as obtained from Fig. 1. The IC_25_ values used were 2 μM, 0.07 μM and 0.04 μM for IMR-5, SHEP and IMR-32 respectively. Data shown indicate percent survival with each drug or in combination compared to corresponding DMSO treatment. Cultures were set up in triplicate and the data presented are representative of two separate experiments. *P* values indicate statistical significance. Figures **a**, **b**, and **c** show findings from the cell lines IMR-32, IMR-5 and SHEP respectively

**Fig. 9:**
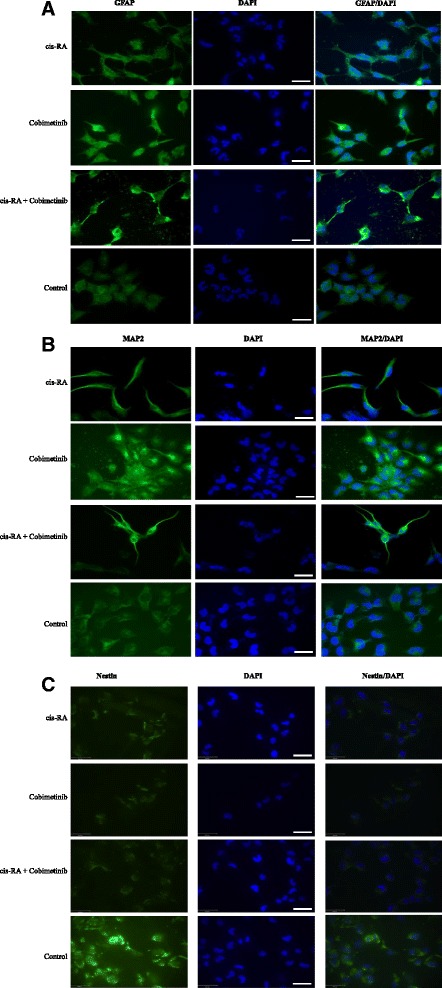
Analysis of cellular differentiation induced by cobimetinib, cis-RA or combination of the two agents. Exponentially growing NB cells were treated with either agent (1 μM cobimetinib or 10 μM cis-RA) or in combination. Cells were then washed and fixed with 4 % paraformaldehyde and permeabilized with 0.05 % Triton X-100. The cells were then evaluated by conventional immunohistochemistry using antibodies to Nestin (1:1000), GFAP (1:1000) and MAP2 (1:800) and fluorescent labelled secondary antibodies. Cells were also counterstained with DAPI and visualized by fluorescence microscopy and photographed. Presented are randomly picked microscopic fields for each experimental condition. Staining for GFAP and MAP2 expression shows an increase and while Nestin expression decreases with differentiation. Changes in morphology with elongated processes are also visible with increased differentiation of the cells. Data are representative of three separate experiments. Scale bar, 34 μM

In the following experiments, we investigated the potential of cis-RA to enhance the activity of cobimetinib in drug combination. Neuroblastoma cells were incubated with increasing concentrations of cis-RA plus IC_25_ concentration of cobimetinib. The IC_25_ values used were 2 μM, 0.07 μM and 0.04 μM for IMR-5, SHEP and IMR-32 respectively. Data presented in Fig. 8a-c indicate that, under the specific experimental conditions used, cis-RA in combination with cobimetinib showed a significant decrease in cell growth in all cell lines tested compared to the drug alone. Next, we explored the induction of differentiation as a potential mechanism in the enhanced activity seen in this combination. For this purpose, the involvement of cells expressing the differentiation markers, GFAP and MAP2 were analyzed using immuno-fluorescence. As can be seen in Fig. 9, there was an increase in GFAP (9A) and MAP2 (9B) expression in cells treated with cobimetinib and cis-RA alone or in combination, compared to control. These data suggest that cobimetinib alone was also able to induce differentiation, which was enhanced after combined treatment with cis-RA. We also tested Nestin to validate differentiation results and noticed a decrease in Nestin expression after the treatment alone or in combination (Fig. 9c).

## Discussion

Neuroblastoma is the most common extracranial solid tumor in the pediatric population and currently, the treatment of high-risk NB with multi-modal therapeutic approaches still results in less than 50 % 5-year event-free survival [15]. Hence, there is a significant and urgent need to develop mechanism based novel therapeutic approaches and early phase clinical trials for the treatment of patients with refractory and high-risk disease. Recently, targeting key receptor tyrosine kinases and their downstream signaling mediators has been shown to be an effective approach in new therapies development in a number of cancer models. In NB, abnormal activation of a number of receptor tyrosine kinases (RTKs) has been reported. These include insulin growth factor 1 (IGF1), c-Kit and the Trk family of receptors. However, as NB cells appear to be highly heterogeneous in the expression of active RTKs, it remains unknown if the targeting individual RTKs would be and an efficient approach. For this reason, the identification of inhibitors for critical downstream signaling nodes that are involved in the transmission of abnormal survival, proliferation and differentiation signals from affected RTKs has been suggested as a potentially viable alternative [16].

The MAPK signaling pathway has been shown to play a critical role in the transmission and coordination of diverse extracellular and environmental stimuli to cell growth mechanisms and MAPK pathway dysregulation has been demonstrated in a variety of human malignancies [17]. MEK1 is a dual-specificity tyrosine threonine protein kinase that occupies a central node in the MAPK signaling pathway [18]. MEK1 phosphorylates ERK1 (p44 MAPK) and ERK2 (p42 MAPK ) to activate pathways that regulate the proliferation and differentiation in cancer cells [19]. A number of previous studies have shown the important role of MEK1 in NB tumorigenesis. For example, in the NB cell line KP-N-RT, IGF-1 leads to phosphorylation of ERK and that the specific MEK1 inhibitor PD98059 leads to the inhibition of IGF-1 mediated cell cycle progression [20]. In this report we aim to investigate the effects of cobimetinib, a potent, orally bioavailable, small-molecule inhibitor of MEK1, against a panel of NB cell lines in order to investigate its feasibility in future therapeutic interventions. Cytotoxicity data presented in Fig. 1 shows effective cell killing, as determined by IC_50_ values, of SK-N-BE(2), SHEP and IMR-32 cells and intermediate activity against SK-N-AS and SK-N-SH cells. The IMR-5 cells appear to be resistant under our experimental conditions. Previously, Eppstein and colleagues have investigated the effects of the MEK inhibitor U0126 against three NB cell lines representing the three types of cells; SK-N-AS (S-type), SH-SY5Y (N-type) and BE(2)-C (I-type) [21]. Their findings showed that although all three cell lines exhibit decrease in pERK with MEK inhibition only the I type cell BE(2)–C had decreased proliferation showing heterogeneity in MEK mediated growth inhibition. Next, we examined the levels of constitutive pMEK in each cell line with the aim of analyzing a potential correlation to their response to cobimetinib (Fig. 2). Interestingly, the IMR-5 cells that appear to have the lowest amounts of pMEK also appear to be resistant to growth inhibition by cobimetinib. This indicates the potential of using constitutive activation status of MEK1 as a biomarker for efficacy in treatment protocols using cobimetinib. However, due to the limited sample size of the cell lines used, studies using a larger cell line panel and/or primary samples are needed to confirm this utility.

To further expand the target modulatory effects of cobimetinib in NB cells, we used antibody arrays to identify changes in various intracellular signaling molecules (Fig. 3). As expected, there is an increase in MEK and decrease in ERK activity was noted. However, we also detected an increase in c-Jun, which is normally a target of ERK activity, and a decrease in the activity of RSK 1/2/3. The RSK family of proteins function downstream of ERK and are considered to be an important conveyer of ERK signaling [22]. In addition we also examined changes in c-RAF, MEK and ERK status in relation to their sensitivity to cobimetinib by evaluating the levels of activation in IMR-32 (sensitive), SHEP (intermediate) and IMR-5 (resistant) cells (Fig. 4). From these data we observed that the dephosphorylation pattern of c-RAF corresponds well with cell growth inhibition as it is most prominent in IMR-32 and next SHEP cells with IMR-5 showing no change. A similar pattern is also seen with ERK activity. However, conversely, IMR-32 cells showed the most increase in MEK activation indicating a consistent pattern of intracellular target modulation and inhibition of proliferation by cobimetinib in distinct NB cell lines. Previously, using the proteomic profiling approach, the potential biological pathways that may confer sensitivity to MEK inhibition in NB subtypes have been investigated by Sandoval and colleagues [23]. Their data suggested that the inhibition of MEK leads to differential intracellular stress response in different NB subtypes and the most resistant cell lines generated unique patterns in protein profiling indicating the utility of these proteins as biomarkers for therapeutic response. In our studies PARP cleavage data provides a biomarker of apoptosis that corresponds to the activity of cobimetinib against the three NB subtypes (Fig. 5). In FACS analysis, cobimetinib treatment has also shown an increased in the number of early apoptotic cells in the IMR-32 and IMR-5 cell line after 24 hour treatment (Fig. 6). We also found that although all three cell lines generated pMEK in response to cobimetinib treatment, the resistant cell line IMR-5 also took the longest time to show this response (30 min to 2 hours), compared to the sensitive cell lines (5 min) (Fig. 7). These data provide dosing and scheduling guidelines for future studies to further investigate the activity of cobimetinib *in vivo*.

Our initial cytotoxicity and target modulation studies described above suggested potential antitumor activity of cobimetinib against the NB cells although there is a range in the sensitivity exhibited by different cell lines. In order to optimize the utility of cobimetinib across all NB subtypes and to enhance the overall effectiveness in future therapeutic regimens, we then explored possible drug combinations that can further enhance the overall effectiveness of cobimetinib against NB. Previous studies have shown that treatment of NB cells with retinoic acid leads to differentiation, cell growth arrest and the decrease in MYCN expression [24]. Cis-RA has also been found to provide survival advantage in combination with immunotherapy with anti-GD2 antibody, combined with GM-CSF or IL-2 [25]. It has been suggested that RA combination with other drugs may lead to synergistic effects allowing the potential to deliver RA at low concentrations to minimize non-specific side effects [24]. Based on this premise we explored the ability of cis-RA to enhance the activity of cobimetinib against the NB cell lines. Interestingly, this combination resulted in increased cell killing in all three cell lines (Fig. 8). Furthermore, morphological and immunohistochemical analysis of the three cell lines treated with this combination showed evidence for morphological differentiation and the expression of markers involved in this process. Unexpectedly we also found that cobimetinib itself is capable of inducing differentiation in NB cells. At present, minimal residual disease is prevented by repeated courses of the RA treatment; however this treatment only improves survival by 35 % in children with metastatic neuroblastoma [26]. In neuroblastoma cell differentiation, cobimetinib alone showed less potency; however, cobimetinib was found to enhance the differentiation induced by RA, suggesting that these compounds could interact cooperatively to improve the NB differentiation and cell killing (Fig. 9). Studies are currently in progress to identify the mechanism of this effect, particularly to understand the influence MEK targeting agents in NB differentiation.

## Conclusion

Taken together, our *in vitro* findings from a panel of NB cell lines suggest that the targeted inhibition of MEK1 by cobimetinib holds the potential to induce potent antitumor activity although there are subsets of cells that may be affected by this treatment. We have also provided key biological markers for this activity that can be used to identify the patient population that may benefit the most by this treatment, Furthermore we also provide evidence for an effective combination of cobimetinib with cis-RA would to enhance antitumor activity in all NB cells. Additional *in vivo* studies in NB xenograft are needed to confirm and further develop these finding for the formulation of effective early phase clinical trials for the treatment of refractory NB patients in the future.

## Abbreviations

NB: Neuroblastoma
MAPKK or MEK: Mitogen-activated protein kinase kinase
cis-RA: cis-Retinoic acid
GFAP: Glial fibrillary acidic protein
MAP2: Microtubule-associated protein 2
ERK1/2: Extracellular signal-regulated kinases
Trk A: Tropomyosin receptor kinase A
Trk B: Tropomyosin receptor kinase B
MAPKs: Mitogen-activated protein kinases
RTKs: Receptor tyrosine kinases
IGF1: Insulin growth factor 1
